# HIV and Black People in Canada: Protocol for a Scoping Review

**DOI:** 10.2196/49066

**Published:** 2023-10-20

**Authors:** Tatyana Graham, Agatha Nyambi, Aisha Barkhad, Maya Stevens-Uninsky, Nadia Rehman, Neera Bhatnagar, Lawrence Mbuagbaw

**Affiliations:** 1 Health Sciences Library McMaster University Hamilton, ON Canada; 2 Department of Global Health McMaster University Hamilton, ON Canada; 3 Biostatistics Unit, Father Sean O'Sullivan Research Centre St Joseph's Healthcare Hamilton, ON Canada; 4 Centre for the Development of Best Practices in Health Yaoundé Central Hospital Yaoundé Cameroon; 5 Division of Epidemiology and Biostatistics Department of Global Health Stellenbosch University Stellenbosch South Africa; 6 Department of Anesthesia McMaster University Hamilton, ON Canada; 7 Department of Pediatrics McMaster University Hamilton, ON Canada

**Keywords:** African, barrier, Black community, Black research, Canada, Caribbean, community, conflict, HIV, mixed methods, public health, qualitative, quantitative, race, racial community, racial, scoping review

## Abstract

**Background:**

Race-based health information is necessary to address disproportionate barriers racial communities face and to achieve optimal health outcomes. In Canada, Black people are disproportionately affected by HIV. There is an emerging body of literature on this topic, but a concise summary is lacking. There is a need to collectively and critically analyze research on HIV in the Black population in Canada to identify knowledge gaps and address this disproportionate burden.

**Objective:**

The aim of this scoping review is to summarize the evidence on HIV and Black people in Canada. The main outcomes of interest are HIV prevalence, access to care, HIV prevention and treatment, the HIV care cascade, and related HIV outcomes. Through this scoping review, we aim to provide a comprehensive overview of the existing literature and highlight topics that need more investigation in future research.

**Methods:**

We will conduct a scoping review of electronic databases using a systematic search strategy for qualitative, quantitative, or mixed methods studies reporting on HIV and Black people in Canada. We will conduct our searches in MEDLINE, Embase, CINAHL, Web of Science, EBSCO, and Google Scholar for literature published between 1985 and 2023. Gray literature, including government reports, dissertations, and other reports, will be included. Search results will be screened, and the full text of relevant literature will be retrieved. The extraction of data will be conducted independently by 2 reviewers. Consensus meetings will be held to resolve conflicts. Our results will be reported according to the PRISMA-ScR (Preferred Reporting Items for the Systematic Reviews and Meta-Analyses Extension for Scoping Reviews).

**Results:**

The initial title and abstract review identified 447 articles. These articles will be critically appraised, and relevant information will be extracted. Information from these articles will be compared using charts and tables. Screening will start in November 2023, and we anticipate publishing the scoping review in June 2024.

**Conclusions:**

The findings from this scoping review will help inform policy, practice, and research on HIV and Black people in Canada.

**International Registered Report Identifier (IRRID):**

PRR1-10.2196/49066

## Introduction

In 2021, it was reported that the HIV epidemic had led to over 80 million people being infected and over 40 million deaths globally [1]. The World Health Organization reported in 2021 that approximately 38.4 million people worldwide are living with HIV [1]. In Canada, 4.5 people per 100,000 were diagnosed with HIV in 2021 [2]. For over 4 decades, the Black community has been disproportionately impacted by HIV [3]. Although the Black community makes up less than 5% of Canada’s total population, 25% of new HIV infections are Black people [3-5]. Additionally, in 2023, Statistics Canada reported higher HIV-related mortality rates among Black Canadians when compared with other ethnicities [6].

In 2014, the Joint United Nations Programme on HIV/AIDS organization, a global initiative aimed at eradicating HIV, developed the 95-95-95 approach (where 95% of people living with HIV are diagnosed, 95% of those diagnosed are on treatment, and 95% of those on treatment have a suppressed viral load) [7]. The Government of Canada aims to end the transmission of HIV by 2030 by adopting the 95-95-95 approach [8,9]. Canada’s action plan includes tracking prevalence, increasing access to HIV care and treatments, and focusing on prevention and the care cascade [3,9]. It is unclear how this goal will be fulfilled in the Black community, although Canada’s Federal Initiative to Address HIV/AIDS aims to address inequities. In 2016, the results of a Canada-wide census illustrated lower educational attainment, housing access, income, and employment in the Black population when compared with the White population [10]. A study by Husbands et al [11] found that Black Canadian participants were less likely to be tested for HIV if they had racist experiences. Systematic disadvantages, lower income, poorer living conditions, higher rates of unemployment, stigma, precarious work, and many other socioeconomic factors plague the Black community and impact the effectiveness of HIV control and care [3,5,12]. These factors also affect Black people’s ability to achieve and maintain viral suppression, which is necessary to end transmission [13].

In addition to behavioral modifications to prevent HIV infection, preventive treatment options for persons at high risk of acquiring HIV include pre-exposure prophylaxis, a combination of antiretroviral drugs [3]. On the other hand, people living with HIV can achieve viral suppression through antiretroviral therapy, therefore decreasing the risk of transmission [3]. Although these options are available, there are still many barriers to accessing them for people in the Black community [8]. A study conducted by Etowa et al [14] identified multilevel barriers, including those at the individual and health system levels that require community-level, health policy, and intersectional interventions. Barriers also remain at other stages of the cascade, such as diagnosis, adherence to antiretroviral therapy, and sustaining care over time [8]. Black French speakers living in predominantly English-speaking provinces also face additional language and communication barriers in HIV diagnosis and treatment [15]. Black people in Canada living with HIV may also be overlooked in surveillance data due to a lack of ethnicity- and race-based data [5]. Therefore, it is beneficial to review the available health literature pertaining to the people living with HIV in the Black community to capture the overall progress toward achieving the 95-95-95 and equitable care.

Some researchers are concerned that once the government of Canada meets the 95-95-95 target, investments in HIV care and research may wane, thus compromising outcomes for the Black community and other subgroups of people who have not met these targets [3]. Identifying the gaps in Black health literature related to HIV is necessary as it will help us better understand the specific vulnerabilities to HIV infection faced by Black people and identify how Black people living with HIV can be better supported. Additionally, having this knowledge can help in the development of a more inclusive HIV response plan and provide the necessary information to fulfill Canada’s Federal Initiative [16].

Although several studies have investigated HIV in Black people, this evidence has not been synthesized in a systematic way, and therefore the state of the evidence is unknown and knowledge gaps have not been thoroughly mapped. This scoping review will highlight areas of HIV research that require further evaluation and identify gaps in the current research regarding HIV and Black people in Canada. A preliminary search was conducted using the Cochrane Database of Systematic Reviews and MEDLINE; no scoping or systematic reviews were found on this topic. The purpose of this scoping review is to inform health policy makers, HIV organizations, and researchers on the state of the evidence on the disproportionate burden of HIV on the Black community in Canada and its impact on their lives.

## Methods

### Overview

This scoping review will use the approach of Peterson et al [17] to inform current HIV research, policies, and education. In contrast to a systematic review, which aims to answer a research question, this scoping review will appraise a wide range of information to illustrate how HIV impacts the Black community in Canada.

This scoping review will be conducted and reported according to PRISMA-ScR (Preferred Reporting Items for the Systematic Reviews and Meta-Analyses Extension for Scoping Reviews) guidelines [18]. The data will be summarized in tables and described narratively. Where possible, we will use means (SD) or counts (percent) to summarize the data from the included studies. Key findings from the studies will be summarized narratively, and the data will be interpreted to identify knowledge gaps, challenges, and opportunities for evidence generation on HIV in the Black community in Canada.

### Collaboration

The public and patients were not involved in this review.

### Eligibility Criteria for Articles

#### Types of Studies

Qualitative, quantitative, mixed methods, experimental, and observational study designs will be considered. Additionally, systematic reviews and gray literature will be considered. To fulfill the eligibility criteria, a study must include data on HIV and Black people in Canada and address an outcome of interest. Literature in English and French will be included, as our reviewers are not fluent in other languages (Textbox 1).

**Eligibility criteria.**

**Inclusion criteria**

- Qualitative, quantitative, mixed methods, experimental, observational studies, systematic reviews, and government reports, dissertations, and gray literature
- Articles in English and French
- Prevalence, access to care, prevention, treatment, the care cascade (initiation of treatment, adherence to medication, and retention in care), and other HIV-related health outcomes

**Exclusion criteria**

- Other articles and websites
- Articles in other languages
- Other topics, whether related to Black people in Canada or not

#### Outcomes

The outcomes of interest are prevalence, access to care, prevention, treatment, the care cascade (initiation of treatment, adherence to medication, and retention in care), and other HIV-related health outcomes.

### Search Strategy

We will conduct a comprehensive and exhaustive search of studies on HIV and Black people in Canada. The search strategy is shown in Textbox 2.

**Search strategy outlining concepts and alternate search terms.**

**Black ethnicity and racial groups**

- minority health/ or exp Minority Groups/ or minority.mp.
- minorit*.mp.
- communit*.mp.
- Africa*.mp.
- Afrique.mp.
- exp Caribbean Region/ or Caribbean*.mp.
- Black or blacks.mp.
- Black canadian*.mp.
- Mixed race*.mp.
- 1 or 2 or 3 or 4 or 5 or 6 or 7 or 8 or 9

**Canadian provinces and territories**

- Canada.tw.
- Canad*.tw.
- 11 or 12
- Newfoundland and Labrador.mp.
- Prince Edward Island.mp.
- lile du prince Edouard.mp.
- Nova Scotia*.mp.
- la Nouvelle-ecosse.mp.
- New Brunswick.mp.
- le Nouveau-Brunswick.mp.
- Quebec*.mp.
- le Quebec.mp.
- Ontario or Ontarian*.mp.
- lOntario.mp.
- Manitoba*.mp.
- le Manitoba.mp.
- Saskatchewan*.mp.
- la Saskatchewan.mp.
- Alberta*.mp.
- lAlberta.mp.
- British Columbia*.mp.
- la Colombie-Britannique.mp.
- Yukon.mp. or Yukon Territory/
- Le Yukon.mp.
- Northwest Territories.mp.
- les Territoires du Nord-Ouest.mp.
- Nunavut.mp.
- le Nunavut.mp.
- Health canada.mp.
- Sante canada.mp.
- CIHR.mp.
- 14 or 15 or 16 or 16 or 17 or 18 or 19 or 20 or 21 or 22 or 23 or 24 or 25 or 26 or 27 or 28 or 29 or 30 or 31 or 32 or 33 or 34 or 35 or 36 or 37 or 38 or 39 or 40 or 41

**HIV**

- HIV Infections/ or HIV-1/ or HIV Antigens/ or HIV Seropositivity/ or HIV Testing/ or exp HIV/ or HIV.mp. or HIV Seroprevalence/ or HIV Seronegativity/ or HIV-2/
- HIV infect*.mp.
- seroconversion/
- 44 or 45 or 46
- 10 and 13 and 42 and 46

### Electronic Search

We will search MEDLINE, Embase, CINAHL, Web of Science, and Google Scholar from 1985 (the date HIV surveillance began in Canada) to 2023 [19]. Key terms will be searched in combination with each other. Some examples include HIV, Black, African, Caribbean, and Canada.

### Reference Lists

The list of references of the included studies will be examined for relevant articles.

### Gray Literature

We will search various government reports, dissertations, and other reports that are significant to this scoping review. Relevant organizations and authors will be contacted.

### Screening

Citations collected from the search will be collated in EndNote [20]. After updating the references and removing the duplicates, we will use the DistillerSR platform (DistillerSR Inc), literature review software, to process the articles [21]. We will first screen titles and abstracts. Only the articles that meet our eligibility criteria will be retained. Articles in languages other than English and French will be excluded, as our reviewers are fluent in those languages. Potentially relevant articles will be downloaded as full text for further screening.

A pilot of the eligibility criteria search strategy will be conducted by 2 reviewers. A sample data collection of 50 abstracts will be done to ensure consistency in the search strategy and approach. Interrater reliability will be measured using the Kappa statistic [22]. The screening will progress when there is at least 80% consensus. The process of screening will be as follows: examining titles and abstracts, analyzing full text for relevance, and then completing data extraction and quality review by 2 reviewers simultaneously.

### Data Extraction

We will extract data from the full text of included articles, including bibliometric information (author, year, and language), study design, province, source of funding, main objectives, participants, sample size, proportion of females, outcome, community involvement, outcomes reported, and key findings. We will pilot our forms on 5% of the data to ensure that they are clear and can be used consistently. All data processing (title and abstract screening, full-text screening, and data extraction) will be conducted by 2 independent reviewers. Disagreement will be resolved by consensus, and if consensus cannot be reached, a third senior reviewer will adjudicate. Agreement will be measured using the Kappa statistic [22]. Data referring to HIV prevalence in the Black community and related statistics will also be extracted. The data extraction framework is shown in Table 1.

**Table 1:** Data extraction framework.

Main category and subcategory			Description
Authors			The institution where authors are based and country of primary affiliation
Lead author			The institution where author is based and country of primary affiliation
Funders			Funder country of origin
Year of publication			The year the research was published
Aims or objectives of study			Stated aim of the study
**Methodology**			
	Study design: subcategory of quantitative, qualitative, or mixed methods	Study design of research within stated category (eg, cross-sectional, ethnography, etc)	
	Population	Eligibility criteria to participate in the study	
	Data collection method	Methods of data collection	
	Data collection and evaluation tools	Types of tools used if applicable and if developed or adapted specifically for this study	
	Outcome measures	Primary and secondary outcomes (where applicable), or selected measures of success (eg, how were objectives of the study measured)	
	Community involvement	Was the community involved in the development of methods or data collection tools (eg, key stakeholder involvement in design), co-designing activities	
	Data analysis	The method of data analysis	
**Results**			
	Reported outcomes	Key HIV outcomes or quantitative or qualitative results related to primary stated outcomes	
	Key findings	Summarize any key findings that report on HIV and Black people in Canada	

### Ethical Considerations

This study does not require human participants. Only secondary data from publicly available sources will be used, and thus ethics approval is not required. The findings will be disseminated through a peer-reviewed manuscript, conferences, abstracts, and an MSc thesis.

## Results

We have identified 447 articles as of May 2023. A critical appraisal of the articles collected will be conducted for the extraction of relevant information. Tables and charts will be used to illustrate similarities and differences in data from various articles. Screening will start in November 2023, and we anticipate publishing the scoping review in June 2024.

## Discussion

### Overview

The current data and health information related to HIV in the Black population in Canada have not been synthesized systematically. There is a need to identify knowledge gaps in order for future research to fill those gaps. This is significant as it can help us understand how and why the Black population in Canada continues to have disproportionately higher HIV infection rates, HIV mortality, and limited access to HIV treatments and care. The lack of HIV health policies and approaches in Canada that are focused toward disproportionately burdened communities, such as the Black community, is concerning. The results of this scoping review will have implications for the development of health policy and how HIV organizations can better support Black people in Canada. As this scoping review is conducted, data will be critically analyzed to ensure that interpretations, comparisons, and implications are reported and concluded accurately.

### Limitations

The Canada-focused approach in this study is the first limitation of this review, as the findings cannot be accurately generalized to global circumstances. However, there is potential for theoretical generalizations to Black communities in countries facing similar dilemmas. A second limitation of this review is that our eligibility criteria exclude articles that are not in English or French. There is a possibility that articles with important information will be left out of our review.

## Abbreviations

PRISMA-ScR: Preferred Reporting Items for the Systematic Reviews and Meta-Analyses Extension for Scoping Reviews

## Data Availability

Data sharing is not applicable to this article as no data sets were generated or analyzed during this study.
